# Sleep mediates the relationship between precarious employment and mental health

**DOI:** 10.1016/j.sleepx.2023.100092

**Published:** 2023-11-13

**Authors:** Saeed Jaydarifard, Simon S. Smith, Kalina R. Rossa, Dwayne Mann, Elahe Nikooharf Salehi, Shamsi Shekari Soleimanloo

**Affiliations:** a Institute for Social Science Research, Faculty of Humanities and Social Sciences, The University of Queensland, Brisbane, Australia; b ARC Centre of Excellence for Children and Families Over the Life Course, The University of Queensland, Brisbane, Australia

**Keywords:** Sleep, Precarious work, Mental health, Stress, Mediation

## Abstract

**Background:**

Current evidence suggests that precarious employment is a risk factor for poor mental health. Although the mechanisms underpinning this relationship are unclear, poor sleep has been proposed to have a role in this relationship. This study explored the mediating effects of poor sleep quality and duration on the relationship between precarious employment and mental health.

**Methods:**

Data were obtained from wave 17 of the Household, Income and Labour Dynamics in Australia survey. A novel precarious employment score (PES) was developed using exploratory and confirmatory factor analyses (CFA) in 8127 workers (4195 female, aged 18–65). Structural equation modelling (SEM) was used to evaluate the mediating effect of sleep quality and duration on the relationship between precarious employment and mental health (SF-36 mental health subscale).

**Results:**

The PES identified 650 workers with a high level of precariousness, 2417 with a moderate level of precariousness, and 5060 workers with a low level of precariousness out of 8127 in total. There was a significant direct association between precarious employment and mental health; with higher precarity increasing the likelihood of poor mental health. The SEM results revealed that sleep quality partially mediated the association between precarious employment and mental health (Coefficient = 0.025, 95 % CI [0.015, 0.034], P ≤ 0.001). However, a mediation effect was not found for sleep duration.

**Conclusion:**

Encouraging precarious employees to improve sleep quality may mitigate the adverse effects of precarious work on their mental health. Further objective measurement of sleep duration warrants a more accurate insight into this mediating effect in this group.

## Highlights

• Developed a novel precarious employment score using factor analyses.
• Most precarious workers were women, younger, single, less educated, and shorter-tenured.
• Higher precarity increases the likelihood of poor mental health.
• Sleep quality mediate the relationship between precarity and mental health.

## 1 Introduction

Precarious employment is defined as a multidimensional construct that includes the characteristics of *employment insecurity*, *income inadequacy*, and *lack of rights and protections* [1,2]. These job characteristics are likely to have mental health consequences for employees [3,4]. The relationship between job precariousness and mental health is well documented, and poor mental health is considered a critically important issue facing precarious workers today [5]. However, the available evidence linking precarious employment with poor mental health is primarily derived from studies that have used only one dimension of precarity (e.g., temporary employment or job instability) to measure mental health outcomes [6]. The relationship between mental health and the multiple dimensions of precarity remains unclear.

One mechanism by which job precarity may influence mental health is through the experience of stress, a common factor in precarious employment [3]. This mechanism has been confirmed by a cross-sectional study of the mediating effects of psychological risk factors (psychological demands, control, and social support) on the association between precarious employment and mental health [7]. Indeed, precarious employment results in a loss of control over future employment and personal lives [8], as well as feelings of uncertainty, unfairness, and powerlessness [3]. Furthermore, anticipating unemployment or perceiving limited opportunities for advancement may induce short-term feelings of irritation, fear, and hopelessness [9]. These feelings may activate a stress response, triggering the release of stress-related hormones [9], and eventually leading to poor mental health [3].

Social and occupational stressors can often extend into nonwork hours, delaying psychological and physiological recovery after work [10,11]. These stressors may be the leading cause of poor sleep [12]. Sleep is a physiological mechanism that is essential for recovery from daily stresses, and thus a prerequisite for effective daily functioning and health [13]. It can be very difficult to obtain sufficient sleep as a precarious worker, due in part to the growing demand for 24/7 work [14], [15], [16], [49]. Consistent with this, a qualitative study on the sleep of precarious workers found insufficient sleep duration due to long or irregular working hours [15]. By contrast, precarious workers with no guaranteed hours of work, such as those in ‘on-call’ care, delivery driving, and gig work, may work fewer hours [17], allowing enough time to achieve sufficient sleep duration.

There is a growing literature on the associations between job precariousness and sleep disturbance. A cross-sectional study [18] examined the effects of employment insecurity on sleep disturbances in 31 European countries using subjective job insecurity to identify precarious workers. In 27 countries, each unit increase in job insecurity increased the likelihood of sleep disturbance by approximately 47 % [18]. Qualitative studies have also consistently documented sleep problems in precarious working conditions, with negative impacts on the workers' mental health [15,19]. In another study, objective features of precarity were not associated with sleep problems, but this association was significant when mediated by subjective assessment of precarity such as perceived job and income insecurity [20]. That is, subjective experience mediates the impact of objective stressors on workers [21]. Precarious employment consists of several characteristics that can operate as stressors, affecting both mental health outcomes and sleep [22,23].

Despite a growing recognition of the sleep problems in the working population, the role of poor sleep in the association between precarious employment and poor mental health is not well described. Sleep is a potentially modifiable behaviour and may provide a point of intervention to improve overall mental health. Current evidence for the impacts of precarity has been primarily drawn from one-dimensional definitions of precarity (e.g., job insecurity or temporariness). Multidimensional precariousness scales demonstrate a more robust pattern of association with health outcomes when compared to traditional one-dimensional approaches [24]. To address these gaps, this study aimed to examine the relationship between sleep disturbances, precarity, and mental health in Australian precarious workers. We hypothesised that higher-precarity workers would experience poorer mental health compared to low and moderate precarity workers. We also hypothesised that short sleep duration and poor sleep quality would mediate the relationship between precarity of job and poor mental health. We tested these hypotheses with a novel multi-dimensional precarity index, using structural equation modelling (SEM).

## 2 Material and methods

### 2.1 Data and participants

Data for the analyses were drawn from Wave 17 of the Household, Income and Labour Dynamics in Australia (HILDA) survey, the largest available panel dataset of Australian households and individuals, comprising nation-wide information on income, housing, employment, health, and wellbeing from 17,570 individuals aged 15 years and over (response rate of 96.7 %). In the present study, the sample was filtered to include individuals of working age (ranging from 18 to 65 years old), which reduced the sample size to 8127 individuals (Mean ± SD age 39.03 ± 13.02 years, 51.6 % female). Human Research Ethics was obtained prior to commencing this study.

### 2.2 Precarious employment score

A novel precarious employment score (PES) was constructed from HILDA items relevant to the definition of precarious employment across the following dimensions: Employment insecurity (4 items), level of income (3 items), and rights and social protections (3 items; see Table 1 below). Cases with at least one missing value in any of the items were removed. Items with a different number of response categories were transformed into values lying between 0 and 3, to give them equal weights. Response options were all categorical or ordinal scales, with higher values being assigned to greater precarity. The arithmetic means of these scores represented the overall PES, ranging from 0 to 30. Consistent with the previous research [25], we used tercile distribution to classify precarious workers at three levels (0 ≥ low precariousness <10; 10 ≥ moderate precariousness <20; 20 ≥ high precariousness ≤30). This classification was used for descriptive analyses and for testing the association between precarious employment and mental health. In addition, a continuous variable of the PES (ranged from 0 to 30) was used for analysing the mediating effect of sleep on precarity-mental health relationship. The scales' validity and reliability were evaluated through factor analyses (exploratory and confirmatory) and Cronbach's alpha coefficients, respectively. In the absence of a scientific gold standard for the allocation of more specific weights, equal weighting is recommended as the most conservative approach [26]. Consistent with this recommended approach and previous precarity measurement scale [27], we applied equal weights to each PES item regardless of the loadings retrieved from CFA. A more detailed description of the construction of the PES is presented in Table 1.

**Table 1:** Operationalisation of the precarious employment score using 10 indicators.

Dimension	Question	Variable treatment
*Employment Insecurity*	Which of these categories best describes your current contract of employment?	<0> if “permanent or ongoing basis” <1> if “Employed on a fixed term contract” <2> if “other (specify)” <3> if “casual”
	Current work schedule (main job)	<0> if “A regular daytime schedule” <1> if “A regular evening shift” | “A regular night shift” <2> if “A rotating shift (changes from days to evenings to nights)” | “Split shift (two distinct periods each day)” <3> if “On call” | “Irregular schedule” | “Other”
	Percent chance of losing job in next 12 months (That is, get retrenched or fired or not have your contract renewed.)	<0> 0%–25 % (not likely; secure) <1> 26%–50 % <2> 51%–75 % <3> greater than 75 % (very likely; insecure)
	Job security satisfaction (0–10)	<0> from 7.6 to 10 (satisfied) <1> from 5.1 to 7.5 <2> from 2.6 to 5 <3> from 0 to 2.5 (unsatisfied)
*Income Inadequacy*	DV: Financial year disposable total income ($) positive values	<0> Greater than 70890.00 <1> from 50101.51 to 70890.00 <2> from 33383.25 to 50101.50 <3> less than 33383.25
	Household income and total income	<0> Greater than 141817 <1> from 103394 to 141817 <2> from 73686 to 1033393 <3> less than 73686
	Prosperity given current needs and financial responsibilities (subjective rating of financial security)	<0> “prosperous” | “very comfortable” <1> “reasonably comfortable” <2> “just getting along” <3> “poor” | “very poor”
*Lack of rights and protection*	Does employer provide paid holiday leave	<0> if “Yes”, <3> if “No”
	Does employer provide paid sick leave	<0> if “Yes”, <3> if “No”
	Trade Union membership	<0> if “Yes”, <3> if “No”

Note. Low scores indicating low precariousness and high scores implying high precariousness. Household and personal income were divided into four quartiles.

### 2.3 Mental health

Worker's mental health was measured using a subscale from the Short Form 36 (SF-36) in HILDA survey [28]. The items assess symptoms of depression and anxiety (‘Have you been a very nervous person?’, ‘Have you felt so down in the dumps that nothing could cheer you up?’, ‘Have you felt downhearted and blue?’) and positive aspects of mental health (‘Have you felt calm and peaceful?’ and ‘Have you been a happy person?’) in the past 4 weeks. Item responses ranged from 1, ‘all of the time’, to 6, ‘none of the time’, which was transformed using Ware, Kosinski [28] method to a 0–100 point scale, with low scores indicating poorer mental health. The SF-36 scale has been validated for use in the Australian population and is widely used in the health literature (see Ref. [29] regarding validation of the SF-36 in the HILDA Survey).

### 2.4 Mediators

**Fig. 1:**
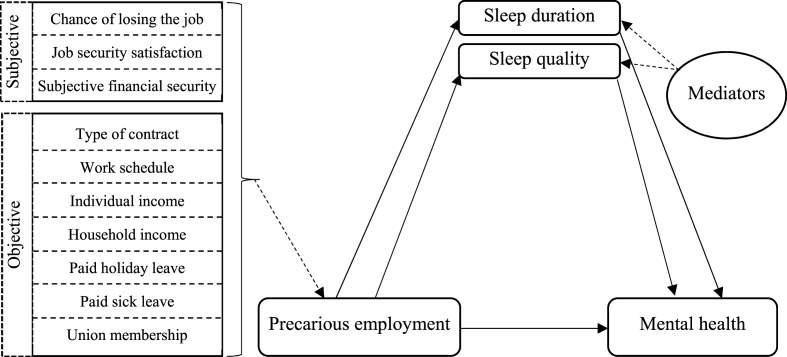
Conceptual model for the effects of precarious employment on mental health. Precarious employment was assessed using both objective (7 items) and subjective (3 items) assessment of precarity. Sleep quality and sleep duration were treated separately as mediators.

Fig. 1 represents the pathways relevant to our research question. Subjective sleep quality was assessed using a single item: “During the past month, how would you rate your sleep quality overall?” with response options ranging from 1 = very good to 4 = very bad. Sleep duration (in hours) was a self-reported item that was calculated separately for the employed and unemployed individuals using a range of questions for work schedule, number of working days, naps, and sleep on workdays, non-workdays, and weekend nights. The questions specifically asked about “How many hours of actual sleep do you usually get?” (From naps or night, on a workday night/non-work night, weekdays and weekends). For employed persons the number of work nights was based on days worked in the main job (not all jobs). A variable for sum of sleep duration excluding time spent napping per week was computed from these responses, and then divided by seven to determine each participant's average sleep duration per night. Implausible values, where individuals slept fewer than 3 or more than 12 h per day (n = 5), were coded as missing data. Sleep duration was analysed as a continuous variable.

### 2.5 Covariates

The covariates were selected based on previous research and theoretical understanding of factors that may confound the association between precarious employment and mental health. These included the participants' gender (male/female), age in years, civil status (married, De facto, separated, divorced, widowed, never married and not De facto), education level (High [Postgraduate - masters or doctorate, Graduate diploma, Graduate certificate, Bachelor or Honours], Intermediate [Advanced diploma, diploma, Certificate III or IV, Year 12], Low [Year 11 and below, Undetermined]), and tenure in current occupation (in years).

### 2.6 Statistical analysis

*Data processing*: All statistical analyses were performed using R statistical software version 4.2.2. Unemployed individuals (N = 6471) and those who did not meet our age criteria (N = 673) were excluded from the analyses. Moreover, incomplete observations for 10 indicators of the PES were removed (N = 2299). Therefore, there was no missing data for the PES. Other variables including the outcomes and mediator had minimal missing data, with no more than 2 % missing data for any variables. Descriptive statistics were calculated as frequencies (%) for categorical variables, whereas means and standard deviations were computed for continuous variables by using “CreateTableOne” function from the “tableone” package in R software, which can be used for both continuous and categorical variables [30]. The distributions of all continuous variables were assessed visually for homogeneity, linearity, and normality and by Anderson-Darling test of the Multivariate Normality Test R package (mvn) at a 0.05 significance level. The Anderson test rejected the normality of data (p values ≤ 0.05). Therefore, data were normalised using standard scaling prior to mediation analyses. Analyses were conducted in two phases including 1) The PES development using exploratory and confirmatory factor analyses; 2) The mediation analyses using structural equation modelling.

*The PES development.* The first step was to evaluate the factorability of the PES for this sample size. Kaiser-Meyer-Olkin (KMO) and Bartlett's tests were performed to quantify whether the 10 indicators of the PES were sufficiently correlated and to determine whether a factor analysis could be performed. A KMO value over 0.5 and a significance level for Bartlett's test below 0.05 suggested a substantial correlation in the data. Cronbach's alpha was also used to test the reliability of the whole data (10 variables) and latent factors. Subsequently, the dataset was randomly split in two halves: a training set for exploratory factor analysis (EFA) and a validation set for confirmatory factor analysis (CFA) to investigate the PES structure. The EFA and CFA models were run via “psych” [31] and “lavaan” [32] R packages. The number of factors was determined through a combination of methods, including the examination of factor loadings, assessment of model fit, consideration of conceptual meanings, and analysis using the parallel analysis, scree plot, and Kaiser criterion. While the Scree plot indicated only one factor, the parallel analysis revealed four, and the new Kaiser criterion (eigenvalues >0.7) indicated two.

Exploratory factor analysis was conducted with a varied number of factors (one, two, three, and four factors) using the maximum likelihood ratio method followed by an oblique rotation (oblimin rotation). Furthermore, polychoric correlations were used for EFA because polychoric correlations are more likely to recover the true factor model than Pearson correlations. It is recommended that EFA be based on polychoric correlations if the ordinal variables are measured by fewer than five to seven categories [33]. Factor loading suggested two factors for the final results, allowing the factors to correlate freely at the latent level. Consistent with the previous studies on the development of the multidimensional measure of precarity, all item loadings in the current study were higher than 0.35, with the exception of household income and subjective financial security (0.219; 0.232, respectively). Thus, the PES included objective and subjective precarity factors with 7 and 3 items, respectively (see Table S2; supplementary materials for factor loadings). A CFA was performed to validate the model supported by the EFA. Several model fit indices were used to assess the adequacy of the model, including Goodness-of-fit indices (GFI), Adjusted goodness of fit (AGFI), Tucker-Lewis Index (TLI), Comparative Fit Index (CFI), Root Mean Square Error of Approximation (RMSEA) and Standardized Root Mean Square Residual (SRMR).

*Association between the PES and outcome variables*. Violin plots were fitted on PES data and outcome variables including mental health, sleep quality and duration for three levels of PES (low, moderate, and high precarious employment) using “ggstatsplot” R package; Non-parametric Kruskal-Wallis H test was used due to the lack of normality in the data to test the significant difference in medians of outcome variables between the three predetermined levels of precariousness. Pairwise comparisons were done by applying the Dunn's Multiple Comparisons *post hoc* test and p-values were adjusted using the Holm adjustment.

*Mediation analyses.* In order to evaluate our hypotheses considering the indirect effect of precarious employment on mental health via sleep quality and duration, we applied structural equation modelling (SEM) using the R package “lavaan” [32]. The significance of the indirect effects was quantified using bootstrapping, with a total of 5000 resamples. Mental health and sleep were reverse-coded for mediation analyses to be interpreted easily. Accordingly, higher scores indicate poorer mental health, sleep, and precarity level. Two models were estimated: a crude model including sleep quality and sleep duration as mediator variables, and an adjusted model accounting for the covariates (age, education, sex, marital status, working hours per week, and tenure in current occupation).

## 3 Results

### 3.1 Descriptive analyses

Characteristics of the studied sample (n = 8127) are shown in Table 2. Gender, age, education, and civil status were significantly related to the level of precariousness (p values < 0.001). There were more women in the high precarity group compared to the group with the low degree of precariousness (58.6 % vs. 48.5 %). Similarly, there was a higher prevalence of younger people (34.8 % vs. 9.3 %), non-partnered (50.6 % vs. 23.3 %), workers with lower level of education (17.1 % vs. 9.7 %), and with lower tenure in their current occupation (5.09 years vs. 10.45 years) in the high precarious group compared to the groups with the lower degree of precariousness (p values ≤ 0.001). Occupational distribution in the study sample can be found in the supplementary materials (Table S1).

**Table 2:** Distribution of worker characteristics by the level of precariousness.

Categorical variables *n (%)*		Precariousness			Total	*P*
		Low	Moderate	High		
n		5060	2417	650	8127	
Sex	Men Women	2604 (51.5) 2456 (48.5)	1059 (43.8) 1358 (56.2)	269 (41.4) 381 (58.6)	3932 4195	≤0.001
Age	18–24 years 25–34 years 35–44 years 45–54 years 55–65 years	469 (9.3) 1373 (27.1) 1174 (23.2) 1198 (23.7) 846 (16.7)	616 (25.5) 646 (26.7) 371 (15.3) 424 (17.5) 360 (14.9)	226 (34.8) 161 (24.8) 93 (14.3) 87 (13.4) 83 (12.8)	1311 2180 1638 1709 1289	≤0.001
Education	Low Intermediate High	493 (9.7) 2419 (47.8) 2148 (42.5)	424 (17.5) 1427 (59.0) 566 (23.4)	111 (17.1) 420 (64.6) 119 (18.3)	1028 4266 2833	≤0.001
Civil status	Partnered Non-partnered	3882 (76.7) 1178 (23.3)	1366 (56.5) 1051 (43.5)	321 (49.4) 329 (50.6)	5569 2558	≤0.001
**Continuous variable *mean (SD)***						
Tenure in current occupation		10.45 (9.98)	6.86 (8.60)	5.09 (7.75)		≤0.001

### 3.2 The PES development

The KMO and Bartlett's tests were applied before EFA. A KMO value of 0.75 and Bartlett's test of sphericity (χ2 = 42742.12, df = 45; p ≤ 0.001) showed satisfactory sample adequacy and factorability of the data. The novel PES included two factors of objective (7 items) and subjective (3 items) precarity, with a reliability coefficient of 0.73. The factor loadings of the items are provided in the supplementary materials (Table S2; supplementary materials). This two-factor structure was confirmed by CFA [(goodness of fit (GFI = 0.980), Adjusted goodness of fit (AGFI = 0.965), Tucker-Lewis Index (TLI = 0.975), Comparative Fit Index (CFI = 0.982), Root Mean Square Error of Approximation (RMSEA = 0.059) and Standardised Root Mean Square Residual (SRMR = 0.039)]. The two-factor model was considered a good fit to our data if GFI >0.95, AGFI >0.90, both CFI and TLI were ≥0.90 = adequate and ≥0.95 = close data-model fit, both RMSEA and SRMR ≤0.10 = acceptable, ≤0.08 = adequate, and ≤0.05 = good data-model fit [34,35].

### 3.3 Precarity and our outcome variables

**Fig. 2:**
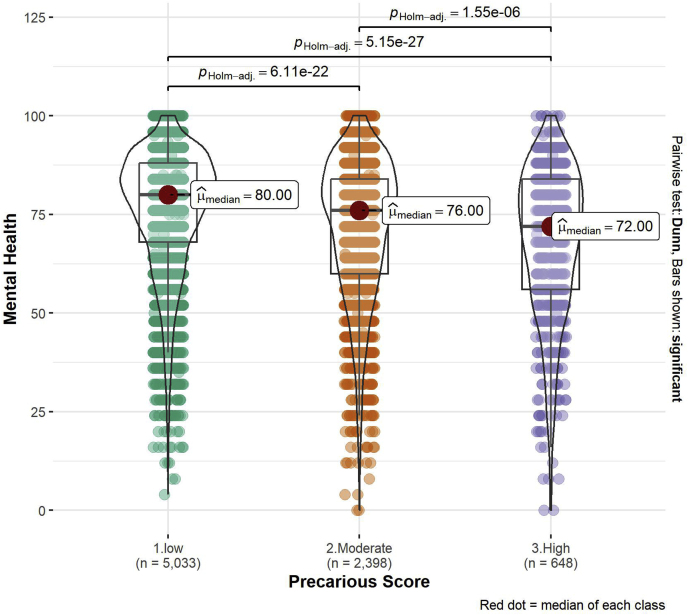
Association between precarious employment and mental health across low, moderate, and high precarious employment. Mental health scale values range from 0 to 100 with low scores indicating poorer mental health. The number of observations for this analysis is 8,079, compared to the entire dataset of 8127 as 48 (0.5 %) observations for the mental health variable were missing.

There was a significant difference between mental health and precarious employment across the three levels of precariousness as observed features (Fig. 2) and assessed with the Kruskal-Wallis H test (H(2) = 178.21, P = 2.00e-39). The *post hoc* pairwise comparisons showed that high precariousness group had poorer mental health (median = 72, mean = 67.77, *P* ≤ 0.001) compared to moderate (median = 76, mean = 71.69, *P* ≤ 0.001) and low precariousness (median = 80, mean = 75.83, *P* ≤ 0.001); with higher precariousness of employment increasing the likelihood of poor mental health.

As shown in Table 3, workers in the high precarious group reported greater feelings of “nervousness”, “downs in the dumps”, “downhearted and blue”, “not calm and peaceful”, and “not happiness” than those in the moderate and low precarious groups (*P* ≤ 0.001). Similarly, individuals in the high precariousness group reported poorer sleep quality than those in the moderate and low precariousness groups (*P* ≤ 0.001). However, there were no significant differences in sleep duration between these three levels of precariousness (P = 0.889).

**Table 3:** Descriptive statistics for outcome variables.

Outcome variables *mean (SD)*	Precariousness			*P*
	Low	Moderate	High	
N	5060	2417	650	
Mental health subscales:				
➢ Been a nervous person	5.00 (1.00)	4.73 (1.15)	4.51 (1.26)	<0.001
➢ Felt so down in the dumps nothing could cheer you up	5.51 (0.86)	5.26 (1.03)	5.05 (1.19)	<0.001
➢ Felt downhearted and blue	4.96 (0.96)	4.75 (1.06)	4.51 (1.16)	<0.001
➢ Felt calm and peaceful	2.99 (1.14)	3.12 (1.20)	3.30 (1.26)	<0.001
➢ Been a happy person	2.53 (1.00)	2.70 (1.08)	2.83 (1.15)	<0.001
Sleep duration (h/min)	7.09 (1.04)	7.11 (1.18)	7.10 (1.28)	**0.889**
Sleep quality	2.13 (0.70)	2.18 (0.72)	2.24 (0.76)	<0.001

Note: The non-significant results are shown in bold text. The p-value was calculated using Kruskal-Wallis test. The Item responses for mental health subscales ranged from 1, ‘all of the time’, to 6, ‘none of the time’. The item responses for sleep quality ranged from 1 = very good to 4 = very bad.

### 3.4 Mediation analyses

**Fig. 3:**
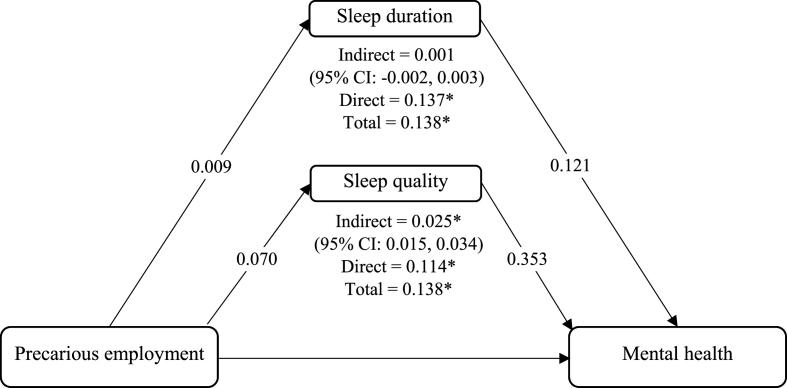
Results of SEM mediation analyses. *P ≤ 0.001.

The results for the direct and indirect effects of precarious employment on mental health with sleep quality and sleep duration as distinct mediators are presented in Fig. 3. The SEM results of crude and adjusted models revealed that sleep quality partially mediated the relationship between precarious employment and mental health (Crude: Coefficient = 0.018, 95 % CI [0.010, 0.025], P ≤ 0.001 & adjusted: Coefficient = 0.025, 95 % CI [0.015–0.034], P ≤ 0.001), which indicates that higher levels of precariousness increased the probabilities of having poor mental health through poor sleep quality. However, sleep duration did not mediate the relationship between precarious employment and mental health (Crude: Coefficient = −0.000, 95 % CI [−0.002, 0.002], P = 0.912 & adjusted: Coefficient = 0.001, 95 % CI [−0.002, 0.003], P = 0.462).

## 4 Discussion

This study explored the potential mediating effects of sleep quality and duration on the relationship between precarious employment and mental health, in a representative sample of Australian workers. The findings revealed that precarious employment, defined by a multidimensional index, is more prevalent among certain sociodemographic groups. Precarious employment was associated with an increased prevalence of experiencing symptoms of poor mental health. Finally, the results revealed that sleep quality but not sleep duration partially mediated the effects of precarious employment on mental health in a representative sample of Australian workers.

Descriptive analyses revealed that being younger, female, not having a partner, having less working experience, and having a lower degree of education increased a worker's susceptibility to high precariousness. These findings are consistent with previous studies [36,37], which have shown that workers with similar characteristics often face lower wages, low-skilled work, less job security, limited benefits, and opportunities for advancement.

Consistent with previous studies that used multidimensional measures of precarious employment in Europe [[38], [39], [40]], our findings identified a significant association between precarious employment and poorer mental health in Australia. This finding supports the view that precarious employment is a genuinely multifactorial phenomenon that is unlikely to be adequately captured by unidimensional indicators [41]. One explanation for the association between precarious employment and poor mental health is that the lack of job and economic security, rights, and social benefits impacts mental health through both objective and subjective experiences. This possibility is supported by the present study, which used seven objective and three subjective indicators of precarity. Understanding the subjective perceptions of individuals towards their precarious work provides a more comprehensive understanding of the relationship between precarity and mental health [42,43]. It is likely that an individuals’ subjective experiences of their employment contributes to their psychological state [20]. While objective indicators can provide helpful knowledge on the structural characteristics of employment (e.g., job security, benefits, and rights), they may not fully represent the subjective experiences and psychological impact of precarity.

The findings of current study provide new information about the mechanisms that link precarity to mental health [44]. We confirmed that work precarity may impact the quality of sleep, which in turn decreases the worker's mental health. Certain characteristics of precarity may trigger stress in workers, where stress is known risk factor for poor sleep [45]. Perceived job insecurity and the lack of stability associated with precarious employment can increase stress, which makes it challenging for individuals to sleep well [20]. Financial difficulties, poor working time arrangements, and limited access to benefits may further contribute to sleep disturbances among individuals with precarious employment. Consequently, precarious workers with poor sleep quality may be at risk of poor mental health. Promoting sleep quality along with improvements in working conditions for precarious workers may be effective in improving their mental health.

Although a few studies have examined sleep quality in the context of precarity [15,[18], [19], [20]], this is the first study to investigate the relationship between sleep duration and precarious employment. In the current study, participants obtained an average of 7.10 h of sleep per night, which meets the recommended amount of sleep [46]. The results revealed that sleep duration did not mediate the relationship between precarious employment and mental health. Our findings suggest that a public health message may need to shift from “did you get enough sleep?” to “did you sleep well?”. One possible explanation for this may be that the subjective experience of poor sleep quality, even with sufficient sleep duration (e.g., 7.10 h per night in our study), may have a stronger association with mental health issues. It has been shown that individuals who reported insomnia with normal/non-disturbed sleep as measured by polysomnography exhibited more mental health issues such as depression and anxiety than those who reported good sleep quality with objectively disturbed sleep [47]. Another possible explanation for the lack of a significant mediating effect for sleep duration could also be influenced by methodological factors, such as lack of objective assessment of sleep duration. Furthermore, a lower percentage of the occupations in our sample (6.4 %; Table S1 supplementary materials) were likely to operate on a 24-h/day schedule with an increased risk of short sleep duration, such as transportation and warehousing [48]. Further research employing a variety of occupations are needed to better understand sleep duration in precarious workers and identify subgroups for intervention.

### 4.1 Strengths and limitations

Some limitations should be considered while interpreting the findings of this study. The most important limitation of this study concerns using cross-sectional data. Although we found evidence for a relationship between job precariousness and poorer mental health, we were unable to establish causal mechanisms for this relationship. For example, it is possible that workers with previous mental health problems, such as depression, are more likely to gain precarious employment, which may have implications both for how they perceive that employment (whether it is secure or not), and for sleep quality. Therefore, further longitudinal studies are needed to further examine these relationships. Although our dataset is quite comprehensive, it is important to acknowledge that the reliance on self-reported data and interview procedures involves the possibility of method variance, which may have resulted in bias. The association and mechanisms between poor sleep and precarious employment need to be further elaborated and corroborated by employing prospective and objective data.

Despite these limitations, this study makes several novel contributions to the existing literature. First, we developed a multidimensional measurement scale consistent with the definition of precarious employment to identify precarious workers in Australia using a national dataset. Furthermore, we used both objective and subjective measures of precarity to identify precarious workers, providing a comprehensive knowledge of the association between precarity and mental health. Second, despite a growing recognition of the consequences of sleep problems, particularly for the working population, research on sleep problems has been limited in precarious employment. To our knowledge, no-one has analysed the links between the multidimensional precarious employment and sleep duration, which is crucial for precarious employees due to their unpredictable schedules. Finally, a novel contribution of this study was to examine two new potential mediators of precarious employment, sleep quality and duration, on mental health in Australian context.

## 5 Conclusions

This study found that work precarity is robustly associated with poor mental health in a large population of Australian precarious workers. Findings also revealed the mediating effects of sleep quality on the association between precarious employment and mental health. However, no association was observed using sleep duration as a mediator. This suggests that even with sufficient sleep duration, perceived poor sleep quality may have a stronger association with mental health issues. Further objective measurement of sleep duration is required for a better understanding of the role of this factor in worker's mental health. Future research could look at the causal relationship between these variables, follow the job trajectories of the workers, and continue to develop the PES. Strategies to improve sleep quality for precarious workers should be trialled.

## Funding details

This research did not receive any specific grant from funding agencies in the public, commercial, or not-for-profit sectors.

## Disclosure of interest

The authors report no conflict of interest.

## Declaration of competing interest

The authors declare that they have no known competing financial interests or personal relationships that could have appeared to influence the work reported in this paper.
