# Supplementary material for: Sleep mediates the relationship between precarious employment and mental health
Source: Sleep Med X. 2023 Nov 13;6:100092. doi: 10.1016/j.sleepx.2023.100092 (PMC10694582; doi:10.1016/j.sleepx.2023.100092)
Supplement: Multimedia component 1 [file mmc1.docx]

**Supplementary materials**

Sleep mediates the relationship between precarious employment and mental health

**Table S1.** Occupational distribution in the study sample by the level of precariousness

**Table S2.** Factor loadings from exploratory factor analysis of the PES

This supplementary material has been provided by the authors to give readers additional information about their work.

**Table S1.** Occupational distribution in the study sample by the level of precariousness

| **Occupation**  ***n (%)*** | **Precariousness** | | | **Total** |
| --- | --- | --- | --- | --- |
|  | **Low** | **Moderate** | **High** |  |
| n | 5060 | 2417 | 650 | 8127 |
| Professionals | 1656 (32.7) | 398 (16.5) | 64 ( 9.8) | 2118 (26.1) |
| Clerical and Administrative Workers | 736 (14.6) | 333 (13.8) | 76 (11.7) | 1145 (14.1) |
| Community and Personal Service Workers | 447 ( 8.8) | 458 (19.0) | 178 (27.4) | 1083 (13.3) |
| Managers | 866 (17.1) | 144 ( 6.0) | 13 ( 2.0) | 1023 (12.6) |
| Technicians and Trades Workers | 631 (12.5) | 295 (12.2) | 54 ( 8.3) | 980 (12.1) |
| Sales Workers | 248 ( 4.9) | 293 (12.1) | 89 (13.7) | 630 ( 7.8) |
| Labourers | 200 ( 4.0) | 298 (12.3) | 129 (19.8) | 627 ( 7.7) |
| Machinery Operators and Drivers | 273 ( 5.4) | 197 ( 8.2) | 47 ( 7.2) | 517 ( 6.4) |

**Table S2.** Factor loadings from exploratory factor analysis of the PES.

| Variables | Factor loading | | Cronbach's alpha |
| --- | --- | --- | --- |
|  | Objective (factor 1) | Subjective (factor 2) |  |
| Type of contract | 0.935 |  |  |
| Work schedule | 0.441 |  |  |
| Individual income | 0.617 |  |  |
| Household income | 0.219 |  | 0.75 |
| Paid holiday leave | 0.987 |  |  |
| Paid sick leave | 0.987 |  |  |
| Union membership | 0.433 |  |  |
| Chance of losing the job |  | 0.832 |  |
| Job security satisfaction |  | 0.878 | 0.50* |
| Subjective financial security |  | 0.232 |  |

* Cronbach alpha values are quite sensitive to the number of items in the scale. With short scales (e.g., 3 items for second factor), it is common to find quite low Cronbach values.
